# Potential fumigant toxicity of essential oils against *Sitotroga cerealella* (Olivier) (*Lepidoptera: Gelechiidae*) and its egg parasitoid *Trichogramma evanescens* (*Hymenoptera: Trichogrammatidae*)

**DOI:** 10.1038/s41598-024-56611-3

**Published:** 2024-03-15

**Authors:** Huda H. Elbehery, Samar S. Ibrahim

**Affiliations:** Pests and Plant Protection Department, National Research Centre, 33 El-Buhouth Street, Dokki, Giza, 12622 Egypt

**Keywords:** Entomology, Secondary metabolism

## Abstract

*Sitotroga cerealella* is a serious pest of a wide range of stored cereal grains. An essential element of an integrated pest control approach is the application of plant oils as a substitute for chemical insecticides. This study aimed to investigate the fumigant toxicity of *Allium sativum* and *Mentha piperita* essential oils against *S. cerealella* adult moths and the egg parasitoid *Trichogramma evanescens*. Gas chromatography–mass spectrometry analyses detected that Diallyl trisulfide (37.97%) and dl-Menthol (47.67%) as main compounds in *A. sativum* and *M. piperita*, respectively. The results showed that, *A. sativum* at 10.0, 5.0, and 2.5 µL/L air resulted in 100% insect mortality after 24 h exposure. The concentrations of 10.0 and 5.0 µL/L air of *M. piperita* oil resulted in 100 and 96% insect mortality, respectively. The parasitoid adult emergence in the F1 reduced when exposed to LC_99_ of *A. sativum* and *M. piperita* oils by 10.89 and 9.67%, respectively. Also, the parasitism of emerged parasitoid decreased by 9.25 and 5.84% (class I-harmless), respectively. Therefore *A. sativum* and *M. piperita* have the potential to be used as bio-fumigant for the management of *S. cerealella* and can be used alongside the *T. evanescens* in integrated pest management.

## Introduction

Most of the world’s population depends on cereals as a source of carbs, vitamins, minerals, fiber, oils, fats, and protein^1^. Therefore, the explosion of the human population in many developing countries led to an increase in the global demand for major production of cereal crops^2^. Wheat, *Triticum *sp., is a widespread crop that is grown in various parts of the world and can be attacked by a plethora of pests^3^. Beetles and moths are among the insect pests that severely harm wheat crops^4^. The Angoumois grain moth *Sitotroga cerealella* (Olivier) (Lepidoptera: Gelechiidae) is one of the most significant pests of cereal grains such as wheat, corn, barley, and rice throughout the world. The pest's larvae cause injury to wheat grains by feeding on seed contents, which reduces the weight and nutritional value of the grains and makes them more susceptible to diseases. Synthetic chemicals are commonly used to manage *S. cerealella*; however, indiscriminate chemical treatment has a number of negative impacts including increased pest resistance and the spread of secondary pests, as well as negative effects on the environment and human health^5^. As a result, the employment of alternative control techniques has become essential. Finding efficient and eco-friendly alternatives to chemical pesticides presents a significant challenge in limiting the harm wrought by the *S. cerealella* pest and reducing crop losses. Egg parasitoids have been used in biological control far more frequently than other natural enemies, especially those of the genus *Trichogramma* (Hymenoptera: Trichogrammatidae). Also, many research studies recently described plant essential oils as eco-friendly agents to control various pests with the least possible effects on the environment and human health. In order to suppress the population of *S. cerealella* in post-harvest storage, the use of essential oil has been examined as an alternative viable technique for managing this pest. The use of plant essential oils as safe, biodegradable insecticides has gained popularity as a viable substitute for dangerous fumigant pesticides in the fight against stored-product insect pests. Fumigation is a useful technique for controlling pests in stored goods. As a result, numerous researchers investigated the insecticidal activity of essential oils against various stored product pests^6–10^, the findings of their studies suggest that essential oils have potential for the control of stored-product pests. There have been few attempts to study the effect of the essential oils of garlic (*Allium sativum* L.) and peppermint (*Mentha piperita* L.) on the non-target egg parasitoid, *T.* evanescens. Thus, the objective of this study was to examine the potential fumigant toxicity of *A. sativum* and *M. piperita* essential oils against *S. cerealella*, an economically important pest in stored products, and their effects on the non-target egg parasitoid, *T.* evanescens.

## Materials and methods

### Essential oils and their phytochemical screening

Essential oils of *A. sativum* and *M. piperita* were obtained from National Research Centre (NRC), Cairo, Egypt. For Gas Chromatography-Mass Spectrometry (GC–MS) analysis, the samples were dissolved in chloroform at the ratio of 50 µL oil:1 mL chloroform and injected into GC. The GC–MS system (Agilent Technologies) was equipped with gas chromatograph (7890B) and mass spectrometer detector (5977A) at NRC. The GC was equipped with HP-5MS column (30 m × 0.25 mm internal diameter and 0.25 μm film thickness). The carrier gas was helium, which was used at a flow rate of 3.0 mL/min at a split ratio (1:10), an injection volume of 1 µL, and the temperature programme as follows: 40 ºC for 1 min; 10 ºC per minute to 200 ºC and held for 1 min; 20 ºC per minute to 220 ºC and held for 1 min; 30 ºC per minute to 320 ºC and held for 3 min. The injector and detector were kept at 250 ºC and 320 ºC, respectively. By using a spectral range of m/z 30–550 and a solvent delay of 2.5 min, mass spectra were produced using electron ionization (EI) at 70 eV. The quad was 150 ºC warmer than the mass temperature of 230 ºC. By comparing the spectrum fragmentation pattern with those found in the Wiley and NIST Mass Spectral Library data, it was possible to identify various constituents of *A. sativum* and *M. piperita* oils.

### Insects

#### *Sitotroga cerealella*

The *S. cerealella* moths were obtained from naturally infested grains stored in a local warehouse in Cairo governorate, Egypt. The stock culture was maintained for more than 5 generations without exposure to insecticides. The adult moths were reared on intact hard wheat grains inside 1000 mL jars. The resultant moths were collected in special cylinders coated with a wire screen with fine pores that allowed the eggs to pass through and prevent the escape of any moths. In the experiments, the newly emerged adults and freshly deposited eggs were used. Wheat grains were purchased from a local market. In order to kill any insects or parasites present, grains were first stored for about 5 days at − 18 °C before being used. The grains were then adjusted for an additional week before use to the laboratory conditions where the culture was kept. Rearing insects and experiments were carried out under controlled conditions of 28 ± 2 °C, 65 ± 5% RH, and 16:8 (L:D) photoperiod.

#### *Trichogramma evanescens*

The egg parasitoid *T. evanescens* were obtained from the mass rearing of the Laboratory of Biological Control of Insects, Plant Protection Research Institute, Agricultural Research Center, Giza, Egypt. Fresh *S. cerealella* eggs were glued to paper strips (3 × 8 cm). The strips holding *S. cerealella* eggs were exposed to *T. evanescens* adults in glass jars (1000 mL) covered with muslin cloth and tightly tied by a rubber band. Egg strips were renewed daily, and old parasitized eggs were incubated under laboratory conditions. The culture of parasitoid was reared under the previous conditions.

### Fumigant toxicity

#### *Sitotroga cerealella*

The fumigant toxicity of *A. sativum* and *M. piperita* essential oils was examined using *S. cerealella* moth adult stage. Glass jars of 1000 mL capacity provided with their screw lids were used as exposure chambers. Concentrations were carried out by releasing the required amounts of the tested oil (10.0, 5.0, 2.5, 1.25, 0.625 µL/L air) from an automatic micropipette onto Whatman No.1 filter paper disks (2 cm diameter) and were glued on the underside of the screwcap of the glass jar. Ten adults of *S. cerealella* (1–3 days old) were released into each jar supplied with 40 g of wheat before the lid was closed tightly and the cover was sealed with parafilm. For control group, the insects were maintained without oil exposure (0 µL/L air). Five replicates were used for each treatment. Insect mortality was checked after 24 h to calculate lethal concentration values of LC_99_, LC_50_, and LC_25_ for the tested oils.

#### *Trichogramma evanescens*

##### Eemergence rate of *Trichogramma evanescens*

**Figure 1:**
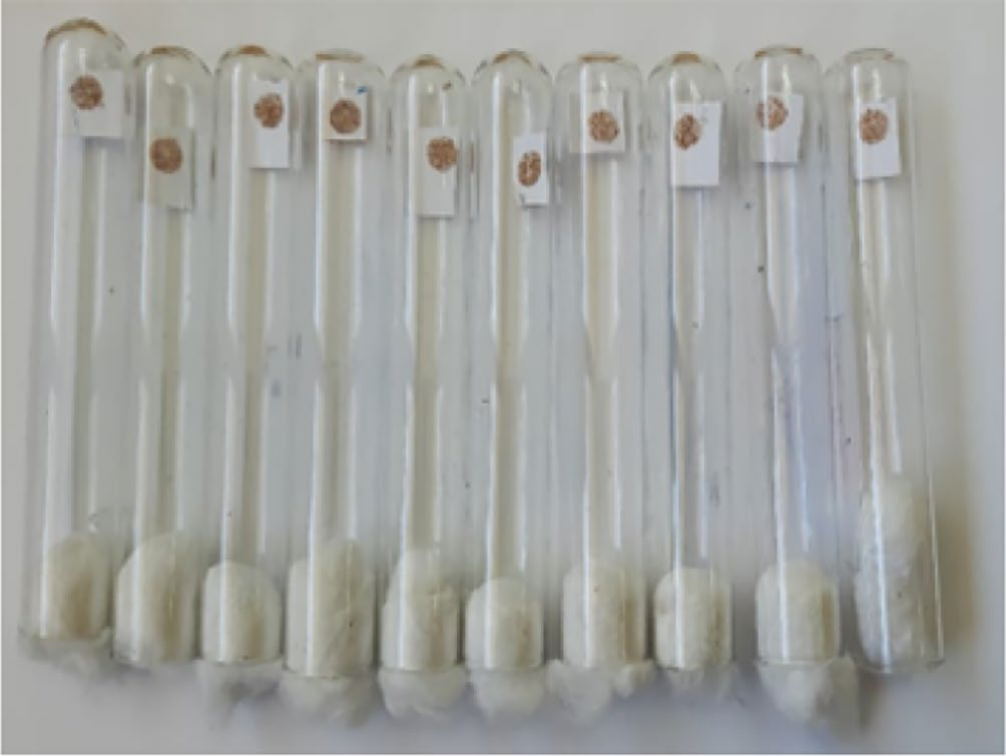
The parasitized *Sitotroga cerealella* eggs strip (1 mm diameter) kept separately inside the glass tubes closed with cotton plug.

**Figure 2:**
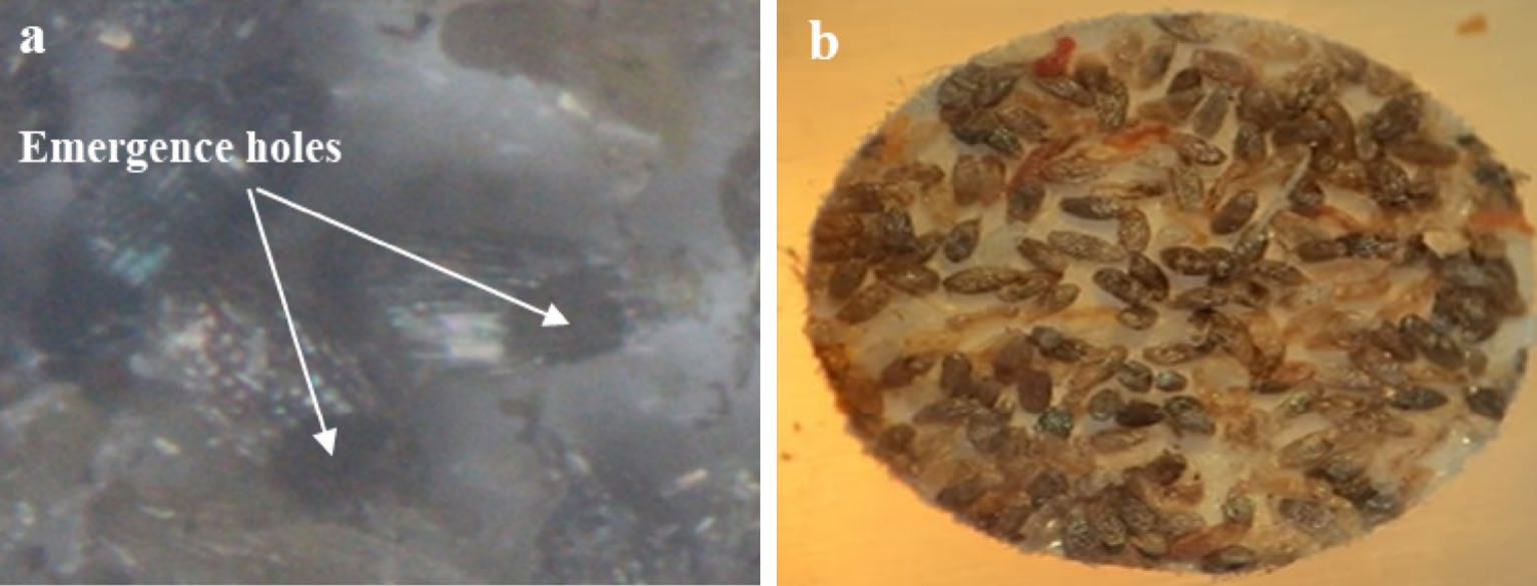
Image of *Sitotroga cerealella* eggs showing, (**a**) emergence holes of parasitized *S. cerealella* eggs, and (**b**) black parasitized *S. cerealella* eggs with *T. evanescens*.

To evaluate the fumigant toxicity of *A. sativum* and *M. piperita* essential oils on the emergence rate of the adult parasitoid, an equal number of 1-day-old *S. cerealella* eggs (110 ± 10) for each treatment were dispersed on self-adhesive strip (1 mm diameter) and exposed to adult parasitoid for 24 h. After 4 days the eggs became black (i.e., clear evidence of parasitism), then the parasitized egg strips were kept in glass jars. Egg strips were exposed to the previously estimated LCs of essential oils vapor on *S. cerealella* (LC_99_, LC_50_, and LC_25_) for 24 h as described previously. After exposure, the egg strips were placed separately in glass test tubes (Fig. 1), and the number of emergence holes (Fig. 2a) were counted to evaluate the percentage of adult emergence.

##### Parasitism rate of emerged *Trichogramma evanescens* (F1)

This experiment was performed to estimate the efficiency of emerged *T. evanescens* adults (F1) of previous experiment. Freshly mated parasitoid (ten adults/each egg strip) were released in glass jars (1000 mL) containing *S. cerealella* eggs strips (110 ± 10). After 24 h of exposure to the parasitoids, *S. cerealella* egg masses were removed from the jars and put into individual transparent test tubes. The number of darkened eggs was counted under binocular to determine the parasitism% (Fig. 2b). As a control, the individuals were maintained in test tubes without any fumes. For each treatment, the studies were repeated ten times.

The International Organization for Biological and Integrated Control (IOBC) proposed four toxic classes: I = harmless, II = slightly harmful, III = moderately harmful, and IV = harmful. These classes are based on the reduction of *T. evanescens* generation emergence or parasitism by < 30%, 30–79%, 80–99%, and > 99%, respectively^11^.

The proportion reduced emergence and parasitism rates were computed as^12^:

1 $$100-mean\left[\left(\frac{{\%}mean \; of \;the \;treatment}{{\%}mean \;of \;the \;control}\right)*100\right]$$

### Data analysis

The mortality, emergence rate, and parasitism rate results were analyzed using One-Way ANOVA, and Duncan's test was used to determine whether there were significant differences between the treatments (P < 0.05). Average mortality data for each treatment were subjected to probit analysis^13^ to obtain the lethal concentration (LC) values. LC values were considered significantly different at the P < 0.05 level. Data analyses were performed using SPSS version 18.0^14^.

## Results

### GC–MS analysis

**Figure 3:**
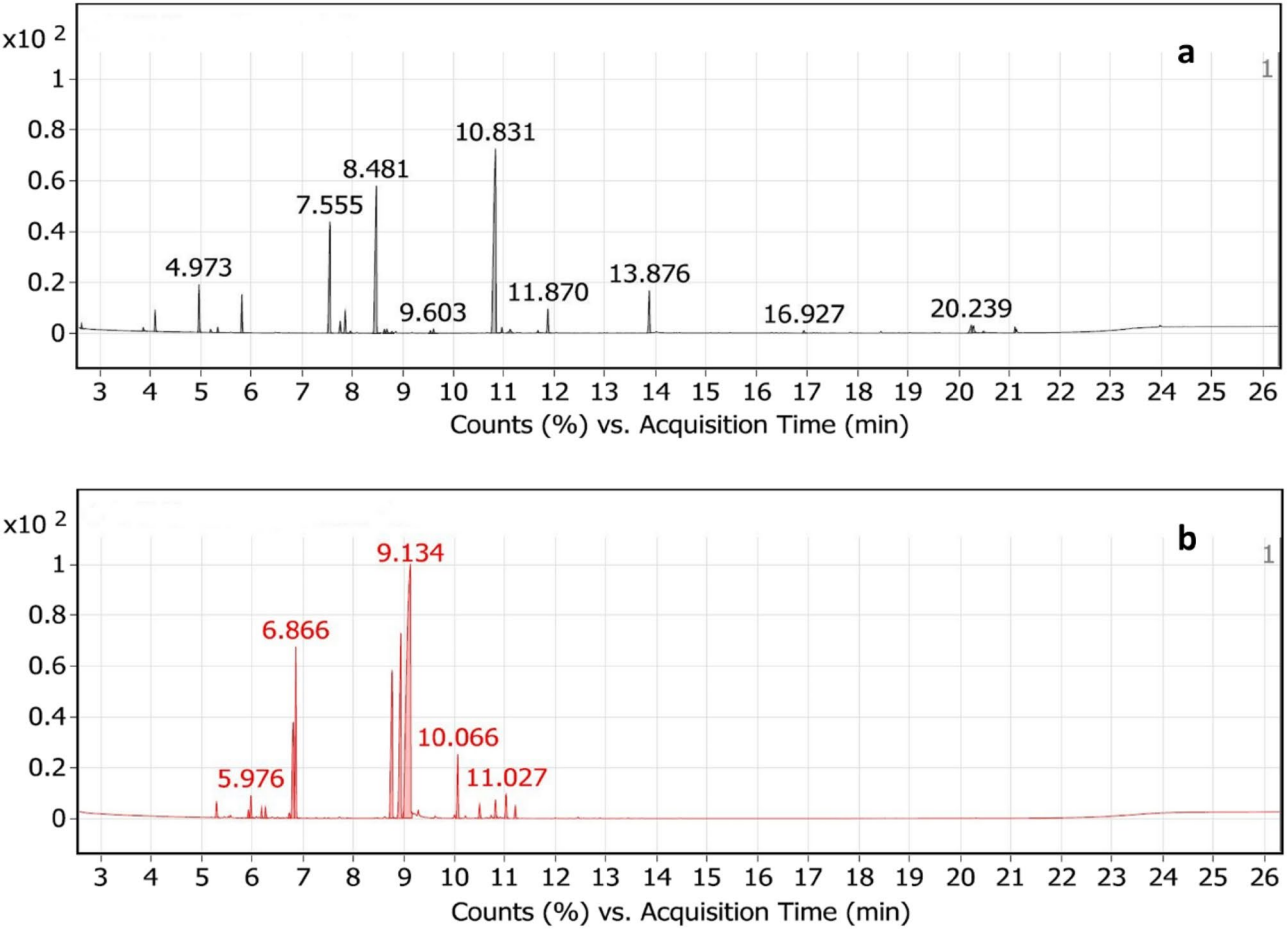
GC–MS chromatogram of (**a**) *A. sativum*, and (**b**) *M. piperita* essential oils.

A total of 28 compounds were detected in *A. sativum* (Table 1, Fig. 3a). The major detected compounds were Trisulfide, di-2-propenyl (Diallyl trisulfide) (37.97%), Trisulfide, methyl 2-propenyl (Allyl methyl trisulfide) (21.65%), and Diallyl disulphide (13.7%). The other sulfur-containing compounds identified in *A. sativum* were Diallyl tetrasulphide (4.47%), Dimethyl trisulfide (3.33%), Allyl methyl disulfide (2.34%), Diallyl sulfide (1.61%), Dimethyl tetrasulphide (0.38%), Trisulfide, allyl propyl (0.44%), and Trisulfide, methyl propyl (0.32%). Table 2 and Fig. 3b show the chemical composition of *M. piperita* essential oil, with dl-Menthol (47.67%), Cyclohexanone, 5-methyl-2-(1-methylethyl)- (15.38%), and l-Menthone (10.82%) being the main constituents. Also, Eucalyptol, d-Limonene, and (−)-Carvone were detected in percentages of 9.12, 7.0, and 3.28%, respectively. Whereas, 1R-MENTHYL ACETATE, beta-Pinene, and alpha-Pinene were detected at percentages of 1.22, 1.03, and 0.71%, respectively.

**Table 1:** GC–MS analysis of *A. sativum.*

RT^a^	Compound	Area^b^	Area sum^c^ %
2.646	Disulfide, dimethyl	1,542,477.74	0.46
3.869	1,2-Dithiolane	1,303,091.32	0.39
4.101	Diallyl sulfide	5,362,375.43	1.61
4.973	1,3-Dithiane	12,872,348.11	3.87
5.199	(E)-1-Methyl-2-(prop-1-en-1-yl)disulfane	955,114.85	0.29
5.341	(Z)-1-Methyl-2-(prop-1-en-1-yl)disulfane	1,306,146.16	0.39
5.822	Dimethyl trisulfide	11,063,406.52	3.33
7.555	Diallyl disulphide	45,577,486.06	13.7
7.769	(E)-1-Allyl-2-(prop-1-en-1-yl)disulfane	4,089,068.35	1.23
7.864	(Z)-1-Allyl-2-(prop-1-en-1-yl)disulfane	6,554,939.48	1.97
7.965	Disulfide, 1-methylethyl propyl	722,537.79	0.22
8.481	Trisulfide, methyl 2-propenyl	72,038,201	21.65
8.635	Trisulfide, methyl propyl	1,057,660.12	0.32
8.683	4-Methyl-1,2,3-trithiolane	1,253,071.29	0.38
8.784	(Z)-1-Methyl-3-(prop-1-en-1-yl)trisulfane	726,294.57	0.22
9.543	3-Vinyl-1,2-dithiacyclohex-5-ene	741,520	0.22
9.603	Tetrasulfide, dimethyl	1,271,825.59	0.38
10.831	Trisulfide, di-2-propenyl	126,327,378	37.97
10.956	1-Allyl-3-propyltrisulfane	1,479,561.95	0.44
11.128	(E)-1-Allyl-3-(prop-1-en-1-yl)trisulfane	2,142,487.63	0.64
11.668	5-Methyl-1,2,3,4-tetrathiane	911,030.7	0.27
11.87	Disulfide, methyl 2-propenyl	7,795,931.8	2.34
13.876	Tetrasulfide, di-2-propenyl	14,882,786.74	4.47
16.927	1-Allyl-3-(2-(allylthio)propyl)trisulfane	842,951.31	0.25
20.239	Linoelaidic acid	4,125,808.49	1.24
20.28	Oleic acid	3,409,562.2	1.02
20.482	9,12-Octadecadienoic acid (Z,Z)-	797,992.11	0.24
21.1	Isopropyl linoleate	1,535,153.59	0.46

^a^RT: retention time.

^b^Area: area of the peak.

^c^Area sum: peak area/total peak area.

**Table 2:** GC–MS analysis of *M. piperita.*

RT^a^	Compound	Area^b^	Area sum^c^ %
5.299	alpha-Pinene	4,610,910.28	0.71
5.929	Sabinene	2,102,034.93	0.32
5.976	beta-Pinene	6,685,768.24	1.03
6.19	beta-Myrcene	3,126,726	0.48
6.267	3-Octanol	3,268,106.99	0.5
6.736	p-Cymene	1,729,174.07	0.27
6.813	d-Limonene	45,355,728.66	7
6.866	Eucalyptol	59,100,967.28	9.12
8.766	l-Menthone	70,068,131.22	10.82
8.944	Cyclohexanone, 5-methyl-2-(1-methylethyl)-	99,607,862.3	15.38
9.134	dl-Menthol	308,781,766.4	47.67
10.066	(−)-Carvone	21,249,373.86	3.28
10.499	Cyclohexanol, 5-methyl-2-(1-methylethyl)-, acetate, (1.alpha.,2.beta.,5.beta.)-	4,370,556.15	0.67
10.808	Cyclohexanol, 5-methyl-2-(1-methylethyl)-, acetate	6,004,556.41	0.93
11.027	1R-MENTHYL ACETATE	7,901,313.44	1.22
11.205	Cyclohexanol, 5-methyl-2-(1-methylethyl)-, acetate, (1.alpha.,2.alpha.,5.alpha.)	3,758,196.8	0.58

^a^RT: retention time.

^b^Area: area of the peak.

^c^Area sum: peak area/total peak area.

### Toxicity study

**Figure 4:**
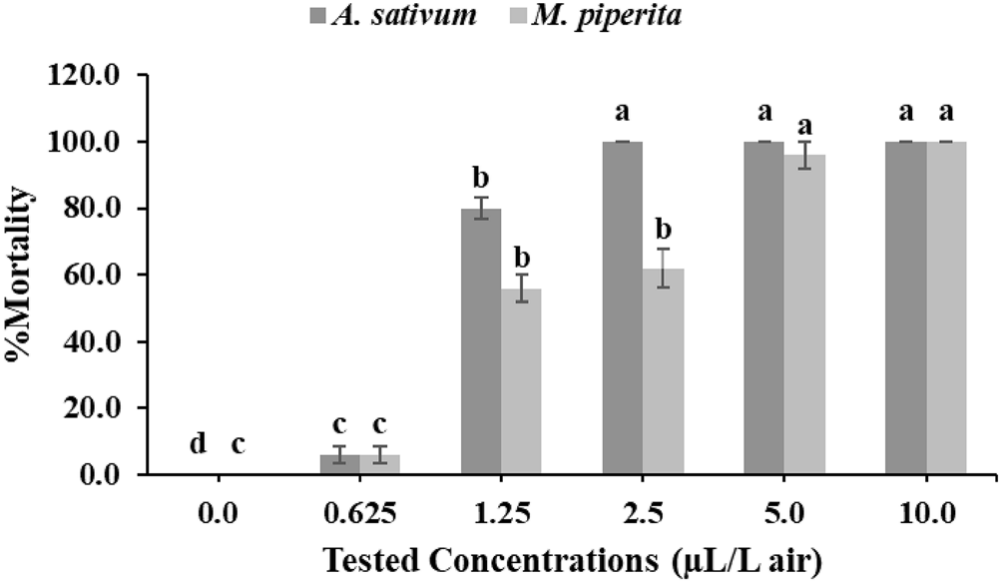
Mortality of *Sitotroga cerealella* adult moths after 24 h exposure to *A. sativum* or *M. piperita* oil. Mean (± SE) values with different letters within the same oil treatment are significantly different (P < 0.05) (ANOVA) (Duncan test).

Fumigant toxicity of *A. sativum* and *M. piperita* oils against *S. cerealella* adult moths is illustrated in Fig. 4. The mortality rate increased as the oil concentration increased after a 24-h exposure. The highest percentage of insect mortality (100%) was observed for *A. sativum* oil concentrations of 10.0, 5.0, and 2.5 µL/L air. The mortality rate decreased to 80% following 1.25 µL/L air *A. sativum* fumigation. In contrast, only 6.0% of insects died when *A. sativum* was applied at the lowest concentration (0.625 µL/L air). Comparably, higher *M. piperita* oil concentrations (10.0, 5.0 µL/L air) led to a higher mortality rate of 96% and 100%, respectively. Also, 2.5 and 1.25 µL/L air caused mortality percentage > 50% after 24 h exposure (Fig. 4). Toxicity data obtained in Table 3 indicated that LC_25_, LC_50_, and LC_99_ of *A. sativum* oil against *S. cerealella* adult moths after 24 h exposure was 0.82, 1.04, and 1.80 µL/L air, respectively. The LC values resulted after fumigation with *M. piperita* oil were 0.79, 1.88, and 5.65 µL/L air for LC_25_, LC_50_, and LC_99_, respectively.

**Table 3:** Toxicity of *A. sativum* and *M. piperita* oil on *Sitotroga cerealella* adult moths after 24 h exposure.

	*A. sativum*	*M. piperita*
LC_25_ (confidence limits µL/L air)	0.82 (0.67–0.92)	0.79 (0.26–1.17)
LC_50_ (confidence limits µL/L air)	1.04 (0.94–1.15)	1.88 (1.55–2.23)
LC_99_ (confidence limits µL/L air)	1.80 (1.59–2.19)	5.65 (4.78–7.12)
Slope ± SE	3.067 ± 0.50	0.618 ± 0.08
Chi^2^	4.27	35.01
Degree of freedom (df)	23	23
P-value	0.000	0.000

### Effect of *A. sativum* and *M. piperita* essential oils on the efficacy of *T. evanescens*

Results shown in Table 4 demonstrated that the exposure of parasitized eggs of *S. cerealella* to the fumes of the tested oils affected the emergence rate of the parasitoid. The highest emergence rates of *T. evanescens* were 92.51% and 93.22% at treatment of LC_25_ *A. sativum* and *M. piperita*, respectively. Furthermore, the adult emergence reduced when parasitized *S. cerealella* eggs were exposed to the highest tested concentration LC_99_ of *A. sativum* and *M. piperita* oils by 10.89 and 9.67% (class I-harmless). In the same line, the parasitism rate of the emerged female of F1 generation was affected by *A. sativum* and *M. piperita* (F = 49.042, P < 0.001and F = 44.54, P < 0.001) respectively. When the fresh *S. cerealella* eggs were offered to the emerged F1 females after LC_99_ treatment, the parasitism was decreased by 9.25 and 5.84% (class I), respectively, and these oils were therefore classified as harmless (Table 4).

**Table 4:** Emergence rate, parasitism rate, reduction (E, P%), and toxic class (TC) of *Trichogramma evanescens* from *Sitotroga cerealella* eggs.

Treatments	*Allium sativum*						*Mentha piperita*					
	% Emergence			% Parasitism			% Emergence			% Parasitism		
	Mean ± ES	E%	TC	Mean ± ES	P%	TC	Mean ± ES	E%	TC	Mean ± ES	P %	TC
LC_25_	92.51 ± 0.34 b	2.45	I	91.26 ± 0.58 b	3.53	I	93.22 ± 0.37 b	2.65	I	93.61 ± 0.53 b	2.14	I
LC_50_	90.37 ± 0.51c	4.70	I	87.82 ± 0.44 c	7.16	I	92.07 ± 0.30 b	3.85	I	91.91 ± 0.36 c	3.92	I
LC_99_	84.42 ± 1.09 d	10.89	I	85.84 ± 0.48 d	9.25	I	86.50 ± 0.62 c	9.67	I	90.07 ± 0.29 d	5.84	I
Control	94.83 ± 0.25 a	–	–	94.59 ± 0.33 a	–	–	95.67 ± 0.27 a	–	–	95.66 ± 0.16 a	–	–
F	49.042			69.76			89.23			44.54		
P	0.00			0.00			0.00			0.00		

Means followed by the same letter per column do not differ by Duncan test (P < 0.05).

TC = toxic class, E% = emergence reduction, P% = parasitism reduction.

## Discussion

Plant based insecticides such as essential oils are considered as a promising component for pest control and an alternative to synthetic chemicals. The immediate or long-term implications of insecticides on natural enemies should be considered when integrating synthetic or bio-insecticides with biological control agents to control major agricultural pests^15^. In the current work, the essential oils of *A. sativum* and *M. piperita* effectively caused mortality of *S. cerealella* adult stages. It was observed that the toxicity increased with increasing oil concentration. Earlier studies have mentioned that different essential oils showed an insecticidal impact on stored-product pests^10,16–19^. Our results show that *A. sativum* oil was rich in Diallyl trisulfide (37.97%), Allyl methyl trisulfide (21.65%), and Diallyl disulphide (13.7). Also, *M. piperita* was found to contain dl-Menthol (47.67%), Cyclohexanone, 5-methyl-2-(1-methylethyl)- (15.38%), and l-Menthone (10.82%), as well as Eucalyptol (9.12%), d-Limonene (7.0%), and (−)-Carvone (3.28%). The primary phytocompounds in essential oils vary in quantity and quality according to location and climate. However, the phytoconstituents of garlic essential oil were relatively similar to the analysis reported by earlier studies^20,21^, for instance^22^ who reported that the main volatile compounds of garlic oil were diallyl trisulfide (37.3–45.9%), diallyl disulfide (17.5–35.6%), methyl allyl trisulfide (7.7–10.4%) and the 2-vinyl-1,3-dithiane (3.9–5.9%). Additionally, it was claimed that the three main components of an Egyptian garlic essential oil were diallyl disulfide (25.2%), allyl methyl trisulfide (23.8%), and diallyl trisulfide (21.1%)^23^. Similar to our findings, the major compounds found in *M. piperita* were menthol, isomenthone, and limonene^24^. Previously it was reported that the content of menthol (39.3%) was the highest, followed by menthone (25.2%), menthofuran (6.8%), and menthyl acetate (6.7%)^25^. Also, menthol (68.0%), menthone (9.5%), isomenthone (8.4%), and menthyl acetate (2.4%) were identified as main compounds of *M. piperita* essential oil^26^. However, different production conditions, such as harvest time, location, seasonal factors, and storage time, can cause the components of essential oil in a given plant species to vary^27^. Insect species, exposure method, and phytochemicals are key indicators of a plant's toxic effect^28,29^. The current study suggests that the fumigant toxicity of *A. sativum* and *M. piperita* oils to *S. cerealella* might be attributed to their chemical composition. According to our study, 100% of *S. cerealella* adult mortality was caused by *A. sativum* oil at concentrations of 10.0, 5.0, and 2.5 μL/L air. Furthermore, *M. piperita* caused > 90% mortality at 10.0 and 5.0 μL/L air. Over 50% of the insects died, even at the lower concentration of 1.25 μL/L air of both oils. Similarly, it was reported that *A. sativum* and its two major components, diallyl disulfide and diallyl trisulfide had significant fumigant activity with LC_50_ values at 1.33, 0.99, and 1.02 μL/L air space, respectively, after 24 h exposure against *S. cerealella*^30^. Moreover, *A. sativum* and bags impregnated with *A. sativum* and *Petroselinum crispum* were found to be highly effective against *Corcyra cephalonica*, *S. cerealella*, and *Trogoderma granarium*^31,32^. Previous study demonstrated that *M. piperita* had a significant insecticidal effect against stored product insects; *Sitophilus oryzae*, *Rhyzopertha dominica*, and *Tribolium castaneum*^33^. It was reported that *M. piperita* and *P. nigrum* essential oils had a notable toxicity against *S. oryzae* with LC_50_ values of 85.0 and 287.7 µL/L air, respectively, after 72 h exposure^24^. Earlier study indicated that *M. piperita* oil showed significant fumigation toxicity against *T. castaneum*, *Lasioderma serricorne*, and *L. bostrychophila* (LC_50_ = 18.1, 68.4, and 0.6 mg/L air, respectively)^34^. Based on our findings, *A. sativum* and *M. piperita* vapors had an effect on the emergence rate of parasitized *S. cerealella* eggs, the parasitism rate of the adult emerged in the F1 displayed a similar trend. *M. piperita* and *A. sativum* reduced the emergence rate by 10.89 and 9.68% in the parental generation *T. evanescens* when exposed to LC_99_. Likewise, no treatment affected the parasitism capacity of the *T. evanescens* F1 generation by more than 9.25%, which classified the oils as harmless based on the IOBC classification. Fewer studies examined the potential toxicity of *A. sativum* and *M. piperita* essential oils on non-target egg parasitoid *T. evanescens*. Alc´antara-de la Cruz et al.^35^ reported that, the parasitism rate in female *T. galloi* of the paternal generation was decreased by EOs of *Allium sativum*, *Azadirachta indica*, and *Carapa guianensis*. However, our findings were supported by Ercan et al.^36^ who reported that, the eggs of *Ephestia kuehniella* were tolerant to *Prangos ferulacea* essential oil. The thickness, shape, and permeability of the vitelline membrane, among other chorion features, may play a role in the tolerance of the parasitized *S. cerealella* eggs. Since essential oils are a source of biologically active vapors, they can possibly act as effective insecticides, as shown by the reported fumigant activity. The findings indicated that the egg parasitoid *T. evanescens* was relatively unaffected by *A. sativum* and *M. piperita* essential oils, and both oils can be used as eco-friendly, effective bio-fumigant for the control of *S. cerealella*.

## Conclusions

In the current study, the fumigant toxicity of *A. sativum* and *M. piperita* essential oils against *S. cerealella* was evaluated. The toxic effect of plant oils is attributed to the presence of phytoconstituents such as Diallyl trisulfide, Allyl methyl trisulfide, and Diallyl disulfide, as well as dl-Menthol, Cyclohexanone, 5-methyl-2-(1-methylethyl)-, l-Menthone, Eucalyptol, d-Limonene, and (−)-Carvone detected in *A. sativum* and *M. piperita* essential oils, respectively. Both oils resulted in 100% mortality when *S. cerealella* moths were fumigated with 10.0% µL/L air after 24 h of exposure. The toxic fumigant effect of both oils on non-target egg parasitoid *T. evanescens* was also assessed. Three tested concentrations LC_99_, LC_50_, and LC_25_ of *A. sativum* and *M. piperita* slightly affected the adult emergence of *T. evanescens* in F1 and the parasitism%. However, according to the International Organization for Biological and Integrated Control-IOBC's proposed toxic classes, our results indicate that tested oils can be classified as harmless substances to non-target *T. evanescens*. The results of this study therefore suggest that oils from *A. sativum* and *M. piperita* can be used as bio-fumigants to effectively control the *S. cerealella* storage pest without threatening the egg parasitoid *T. evanescens*. However, more studies would be needed to ensure their effectiveness under different climatic conditions.

## Data Availability

All data generated or analyzed during the current study are available from the corresponding author on reasonable request.
